# Combination of anodal tDCS of the cerebellum with a goal-oriented motor training to treat cervical dystonia: a pilot case series

**DOI:** 10.3389/fneur.2024.1381390

**Published:** 2024-04-30

**Authors:** Jean-Pierre Bleton, Charlotte Cossé, Tiphanie Caloc’h, Alcira Suarez Moreno, Elisabeth Diverres, Pascal Derkinderen, Julien Nizard, Jean-Pascal Lefaucheur, Jean-Paul Nguyen

**Affiliations:** ^1^Neurology Department, Clinical Research Department, Hôpital Fondation Adolphe de Rothschild, Paris, France; ^2^Unité de Stimulation Transcrânienne, Clinique Bretéché, Groupe Elsan, Nantes, France; ^3^Service de Neurologie, CHU de Nantes, Nantes, France; ^4^Service Douleur, Soins Palliatifs et de Support et UIC22, Hôpital Laennec, CHU, Nantes, France; ^5^EA 4391, Équipe ENT (Nerve Excitability and Therapeutics), Université Paris-Est Créteil, Créteil, France; ^6^Unité de Neurophysiologie Clinique, Hôpital Henri Mondor, Assistance Publique—Hôpitaux de Paris (AP-HP), Créteil, France

**Keywords:** cerebellar stimulation, cervical dystonia, motor training, neuromodulation, non-invasive brain stimulation, transcranial direct current stimulation, rehabilitation

## Abstract

**Background:**

Transcranial Direct Current Stimulation (tDCS) of the cerebellum shows promise for the treatment of dystonia. Specific motor rehabilitation programs have also been developed in this context. However, the combination of these two approaches has not yet been evaluated to determine their therapeutic potential.

**Methods:**

We report a series of 5 patients with cervical dystonia (CD) poorly controlled by botulinum toxin injections. They were initially treated by a protocol of repeated daily sessions (for 3 or 5 days) of cerebellar anodal tDCS (cer-atDCS) applied alone. In a second time, additional protocols of cer-atDCS were performed in combination with a program of goal-oriented motor training exercises (Mot-Training), specifically developed for the treatment of CD. The clinical impact of the procedures was assessed on the Toronto Western Spasmodic Torticollis Rating Scale (TWSTRS).

**Results:**

Compared to baseline, the maximum percentage of TWSTRS total score improvement was 37% on average after cer-atDCS performed alone (*p* = 0.147, not significant) and 53% on average after cer-atDCS combined with Mot-Training (*p* = 0.014, significant). The TWSTRS pain and functional handicap subscores also improved after the combined protocol. A score of (+3) to (+5) was rated on the TWSTRS response scale after cer-atDCS performed alone or the combined protocol, corresponding to a moderate to striking improvement on dystonia and pain. This improvement lasted longer after the combined protocol than after cer-atDCS alone (3.4 vs. 1.4 months on average, *p* = 0.011).

**Conclusion:**

The combination of cer-atDCS with Mot-Training produced a greater and more prolonged improvement than the application of cer-atDCS alone. Such a combined therapeutic procedure is easy to perform and opens important perspectives in the long-term treatment of CD. These results remain to be confirmed by a randomized sham-controlled trial on a larger sample.

## Introduction

Like other forms of dystonia, cervical dystonia (CD) is marked by an involuntary and inappropriate contraction of certain muscle groups that causes abnormal movements and postures. The main therapeutic strategies are based on the injection of botulinum toxin (BTX), which aims to weaken overactive contracted muscles (agonists) and on physiotherapy, which aims to reinforce the activity of the corrective muscles (antagonists) to improve tonic balance (1). The principle, technique, and results of BTX injections are well known and this treatment is effective in approximately 70% of cases, but the injections must be repeated every 3 months (2). Regarding physiotherapy, a motor training program (Mot-Training) has been developed by Bleton (3) and taken up by other authors (4, 5), which aims to gradually strengthen the activity of the corrective muscles and is well suited for long-term clinical application. The notions of duration, repetition and progressiveness are currently well highlighted in rehabilitation programs for the treatment of dystonia aimed at acting on neuronal neuroplasticity in the medium and long term (6, 7).

Although the cause of dystonia remains unknown, the main pathophysiological mechanism involved lies in a dysfunction within the neural networks connecting the cerebellum, the basal ganglia and the sensorimotor cortex (8). Also, it seems relevant to apply neuromodulation techniques to modulate these networks and correct this dysfunction. In clinical practice, neuromodulation therapy of dystonia is essentially performed invasively, using surgically implanted electrodes and stimulators. Thus, deep brain stimulation (DBS) techniques were developed, mainly targeted at the level of the pallidum and/or the thalamus for the treatment of dystonia. DBS is effective and fully justified in severe forms of generalized dystonia (9). It also seems effective in the context of focal dystonia, including CD (10), but the benefit/risk ratio here is more debatable. On the other hand, there are also non-invasive neuromodulation techniques, mainly repetitive transcranial magnetic stimulation (rTMS) and transcranial direct current stimulation (tDCS). In their therapeutic application for focal dystonia, these techniques (rTMS or tDCS) have mainly targeted the motor or premotor cortex and the cerebellum [see review in Lefaucheur et al. (11)] and have shown some efficacy in the treatment of CD. In particular, a beneficial effect of cerebellar anodal tDCS (cer-atDCS) was reported in two patients with CD (12, 13). Despite positive short-term results, the therapeutic effect was reported to be only transient, requiring repeated stimulation sessions to be maintained (13). By analogy with what has been shown for motor rehabilitation of stroke patients (14), we proposed a combined approach, associating tDCS and physical therapy for the treatment of dystonia. Thus, we aimed at improving and prolonging the therapeutic effect of tDCS by combining repeated sessions of cer-atDCS with a program of physiotherapy (Mot-Training) specifically developed for the management of dystonic patients (3, 15). We report five cases of patients with CD initially treated with cer-atDCS alone then with the combination of cer-atDCS and Mot-Training. The objective of this case series was to show the additional value of combining physiotherapy with non-invasive cortical stimulation for the treatment of CD.

## Methods

### Patients and study plan

All five patients included in this study had reduced efficacy of BTX injections for the treatment of primary idiopathic CD. Study plan includes two phases. The first phase was based on the administration of cer-atDCS protocol alone (three or five daily sessions of 20-min duration within a week). The second phase was based on the administration of cer-atDCS protocol (also three or five daily sessions within a week) combined with Mot-Training. The Mot-Training protocol lasted 20 min, was tailored to the clinical characteristics of each patient (see below) and was performed during the cer-atDCS session (see Supplementary Video S1).

The switch between the first and the second phase was linked to the availability of the physiotherapist when recruited in our center. Thus, patients received the cer-atDCS protocol alone for a variable duration before being able to start with the combined protocol. The duration of follow-up after a stimulation protocol was related to patients’ availability to return to our center and the duration of post-session improvement.

### Clinical assessment

Patients were assessed at the end of each week of stimulation protocol (cer-atDCS alone or combined with Mot-Training) on the French version (16) of the Toronto Western spasmodic torticollis rating scale (TWSTRS) (17). This scale includes three subscores: a severity score (max: 35), a functional handicap score (max: 30) and a pain score (max: 20) for a total score up to 85, with a more elevated score corresponding to a more severe CD. In addition, this scale includes a response scale, ranging from (−1) to (+5). On this scale, a score of (−1) corresponds to a worsening after treatment; a score of (0) corresponds to the absence of worsening or improvement; a score of (+1) corresponds to a minimal or questionable reduction in dystonia and pain without functional improvement; a score of (+2) corresponds to a mild response with some reduction in dystonia and pain and little functional improvement; a score of (+3) corresponds to a moderate response with a noticeable reduction in dystonia and pain and significant functional improvement; a score of (+4) corresponds to a clear response with obvious reduction in dystonia and pain and excellent functional improvement; a score of (+5) corresponds to a striking improvement with little or no dystonia or pain remaining. Additionally, investigators asked patients about the duration of clinical response they subjectively experienced after completion of each protocol.

Statistical analyses were performed with a paired t test after confirming that the data were sampled from a Gaussian distribution and passed the normality test using the method of Kolmogorov and Smirnov. Paired comparisons were made for the various TWSTRS scores between data obtained in the two phases of the study (after cer-atDCS alone or combined with Mot-Training) compared to baseline (before any cer-atDCS protocol). Two sets of data were used, either the values observed after the last treatment session or the lowest value observed during the two phases of the study.

### Cerebellar tDCS

Initially, patients were treated with cer-atDCS alone. We used the HDC kit stimulator (Inomed, Emmendingen, Germany) which allows stimulation by two anodes and one cathode (large square electrodes). The anodes (5 × 5 cm) were placed on each of the two cerebellar hemispheres (1–2 cm below and 3–4 cm lateral to the inion). The cathode (8.5 × 6 cm) was placed on the right supraorbital region. Each cer-atDCS session lasted 20 min with a stimulation intensity of 2 mA. Repeating one session daily for three to five consecutive days over the course of a week has been shown to produce lasting clinical benefit in dystonic patients, especially on CD in our experience.

### Motor training program

As each clinical presentation of CD is unique, there is no standardized physiotherapy rehabilitation program suitable for all patients. Optimal motor training exercises should be selected based on assessment of three-dimensional disorganization of head posture in each patient (18): transverse (torticollis), coronal (laterocollis), or sagittal (retrocollis or antecollis). Disorganization of head posture may be present simultaneously in multiple planes in a patient with CD. For example, a rotational torticollis may be combined with a laterocollis and a retrocollis. They can also be associated with head displacement in the sagittal plane (anterior or posterior shift) or the coronal plane (lateral shift). Postural disorders and disorganization of cervicocephalic movements are linked to the involvement of overactive (dystonic agonists) and underactive (inhibited antagonists) muscles.

Physiotherapy should focus on activating functionally impaired corrective antagonistic muscles (19). Different levels of difficulty of Mot-Training can be proposed according to the severity of their inhibition. First, the head can be turned to the anti-dystonic side in association with facilitation techniques, such as raising the arms or lying on the back, or the head can be rapidly and repeatedly turned as if to say “No.” Second, the head can be voluntarily brought to the anti-dystonic side without using facilitation techniques. Third, the head can be controlled and maintained turned to the anti-dystonic side. Fourth, the movement of the head can be controlled through a full range of motion (from the pro- to the anti-dystonic side) and replaced in a stable position on the midline with the eyes open or closed (proprioceptive control) (20, 21). In this case, exercises should be performed slowly so that they can be controlled from the moment they are initiated to the moment the head returns to the resting position. This prevents the phenomenon of overflow and parasitic co-contractions (22). It usually takes 5–10 s to complete an exercise through its full range of motion. Exercises are interspersed with a rest period equivalent to the duration of the exercise. It has been shown that it was necessary to repeat the exercise for about 20 min to obtain a significant clinical impact (3, 15). Thus, motor training is able to promote neuroplasticity and therefore provide lasting effects over time (23).

An example of Mot-Training program is detailed for patient 1 This woman developed a blepharospasm at the age of 56 and a CD consisting of an antecollis with a left laterocollis a few months later. In this patient, two main deformities were present (Supplementary Video S1, segment 1): (i) left rotational torticollis with active rotation to the anti-dystonic side possible but difficult to control, coupled with compensatory involvement of the shoulder girdle; (ii) an anterior shift of the head in (also called “turtleneck”). Also, according to the control modalities described previously, three tailored Mot-Training exercises were proposed, performed in a seated position, and repeated for 20 min (Supplementary Video S1, segment 2):

- Exercise 1: correction of the anterior dystonic shift by the so-called “double chin” exercise. This exercise consists in moving the head back and placing the chin on the neck (flexion of the lower cervical spine) while keeping the gaze horizontal and straightening up. This exercise reduces cervical hyperlordosis related to dystonic muscle activity (24) by a double movement of extension of the lower cervical spine and flexion of the upper cervical spine. This corrective posture involves the active participation of the deficient extensor muscles of the lower cervical spine (such as levator scapulae, semispinalis and longissimus cervicis muscles) and of the upper cervical spine (such as longus colli et captitis muscles), not in force but in endurance.
- Exercise 2: corresponding to the exercise 1 coupled with a corrective cervical rotation toward the anti-dystonic side (to the right in our patient). This exercise consists of performing a slow and complete cervicocephalic rotation while maintaining the “double chin” posture. This corrective movement involves the active participation of deficient cervical spine rotator muscles (such as obliquus capitis inferior, splenius and sternocleidomastoid muscles).
- Exercise 3: active corrective rotation toward the anti-dystonic side without compensatory movement of the trunk and the shoulder girdle. This exercise consisted in placing the hands behind the head, fingers crossed and elbows apart, before turning the face to the anti-dystonic side and holding this position for 5 to 6 s, before finally replacing the head to a neutral position. This endurance exercise had to be practiced without force to avoid a dystonic reaction.

## Results

All five patients included had primary idiopathic CD, but an additional contribution of neuroleptic-induced dystonia was suspected in patient 4. Main clinical features are presented in Table 1. The patients were four women and one man (patient 3), aged from 40 to 79 years. The duration of CD symptoms ranged from four to 23 years. One patient (patient 3) had refused BTX injection treatment for personal reasons. In the other patients, BTX injections were spaced by 2 months (patient 5), 3 months (patients 2 and 4), or 4 months (patient 1). Overall, BTX injections had lost their efficacy on dystonia (patients 1 and 4) or on pain associated with dystonia (patients 2 and 5) for several months.

**Table 1:** Patients’ demographical data and performed protocol.

	Gender	Age (years)	Disease duration (years)	BTX injection efficacy	Number of cer-atDCS protocols in the first phase	Number of cer-atDCS + Mot-Training protocols in the second phase	Total follow-up duration (months)
Patient 1	F	79	23	Loss of efficacy since 4 months	5 (spaced 3 months apart)	1	22.5 months
Patient 2	F	75	10	Reduced efficacy on pain since 3 months	3 (spaced 1 month apart)	6 (spaced 2 months apart)	17 months
Patient 3	M	63	10	No BTX therapy	2 (spaced 1 month apart)	4 (spaced 3 months apart)	15.5 months
Patient 4	F	40	11	Loss of efficacy since 3 months	1	12 (spaced 2 months apart)	28 months
Patient 5	F	56	4	Reduced efficacy on pain since 2 months	1	8 (spaced 4 months apart)	35 months

BTX, botulinum toxin; cer-atDCS, anodal transcranial direct current stimulation of the cerebellum; Mot-Training, physiotherapy based on motor training exercises.

In the first phase of the study, the cer-atDCS protocol consisted of one daily session on three consecutive days for patients 2 to 5 and on five consecutive days within a week for patient 1. The number of cer-atDCS protocols was only one for two patients, and two to five (spaced 1 to 3 months apart) for the remaining three patients. Clinical assessment, based on the TWSTRS, is presented in Table 2 and was performed at the end of each week of cer-atDCS protocols, except for patient 2 after the second protocol. The TWSTRS total score ranged from 30 to 58 (mean ± standard deviation: 45.3 ± 10.1) at baseline, from 29 to 46 (34.6 ± 6.7) after the last cer-atDCS protocol (*p* = 0.158, paired t test, not significant), and from 15 to 46 (28.6 ± 13.2) after the most efficacious cer-atDCS protocol for each patient (*p* = 0.147, not significant). Thus, the maximum percentage of TWSTRS total score improvement after cer-atDCS performed alone was 37% on average. For example, the improvement on the TWSTRS total score was marked by a 38% decrease in patient 1 (Supplementary Video S1, segment 3). Only the TWSTRS pain subscore significantly improved after the last or the most efficacious cer-atDCS protocol (*p* < 0.01), but not the other TWSTRS subscores. Overall, a score of (+3) to (+5) was rated on the TWSTRS response scale after cer-atDCS protocols, corresponding to a moderate to striking improvement on dystonia and pain. The duration of subjective improvement after completion of a cer-atDCS protocol ranged from 1 to 3 months (1.4 ± 0.9).

**Table 2:** Clinical results assessed on the Toronto Western Spasmodic Torticollis Rating Scale (TWSTRS).

	Baseline	Post-Session 1 (cer-atDCS)	Post-Session 2 (cer-atDCS)	Post-Session 3 (cer-atDCS)	Post-Session 5 (cer-atDCS)	Post-Session 1 (cer-atDCS + Mot-training)	Post-Session 4 (cer-atDCS + Mot-training)	Post-Session 6 (cer-atDCS + Mot-training)	Post-Session 8 (cer-atDCS + Mot-training)	Post-Session 12 (cer-atDCS + Mot-training)
**Severity score (max: 35)**										
Patient 1	22	18	4	3	15	3				
Patient 2	21	4	NA	18		13	NA	15		
Patient 3	13	10	10			7	16			
Patient 4	7	4				8	12	12	NA	8
Patient 5	5	13				11	NA	8	2	
**Functional handicap score (max: 30)**										
Patient 1	23	11	9	4	8	4				
Patient 2	19	10	NA	11		9	NA	8		
Patient 3	20	17	16			11	7			
Patient 4	18	29				12	15	10	NA	15
Patient 5	10	14				8	NA	8	3	
**Pain score (max: 20)**										
Patient 1	13	8	5	8	6	5				
Patient 2	8.5	2	NA	3		7	NA	6		
Patient 3	12	8	5			4	5			
Patient 4	20	13				9	11	8	NA	15
Patient 5	15	8				13	NA	8	9	
**Total score (max: 85)**										
Patient 1	58	37	18	15	29	12				
Patient 2	48.5	16	NA	32		29	NA	29		
Patient 3	45	36	31			22	28			
Patient 4	45	46				29	38	30	NA	38
Patient 5	30	35				32	NA	24	14	
**Response score (−1/+5)**										
Patient 1		5	4	4	4	5				
Patient 2		3	NA	3		3	NA	4		
Patient 3		3	3			3	5			
Patient 4		0				4	3	4	NA	3
Patient 5		0				0	NA	4	5	

cer-atDCS, anodal transcranial direct current stimulation of the cerebellum; Mot-Training, physiotherapy based on motor training exercises.

In the second phase of the study, the cer-atDCS protocol was combined with Mot-Training and was performed on three consecutive days in patients 2 and 4 and on five consecutive days within a week in patients 1, 3 and 5. The number of combined protocols was only one for patient 1, and four to 12 (spaced 2 to 4 months apart) for the remaining three patients. The TWSTRS, was scored at the end of each week of combined protocols, except for patients 2 and 5 after the fourth protocol and for patients 4 after the eighth protocol (Table 2). The TWSTRS total score ranged from 12 to 38 (24.2 ± 11.0) after the last combined protocol (*p* = 0.033, significantly reduced compared to baseline), and from 12 to 30 (21.4 ± 8.3) after the most efficacious combined protocol for each patient (*p* = 0.014, also significant). Thus, the maximum percentage of TWSTRS total score improvement after cer-atDCS combined with Mot-Training was 53% on average. As after the cer-atDCS protocol performed alone, the TWSTRS pain subscore significantly improved after the last or the most efficacious combined protocol (*p* < 0.01), but also the TWSTRS functional handicap subscore (*p* < 0.05). Again, a score of (+3) to (+5) was rated on the TWSTRS response scale after combined protocols, corresponding to a moderate to striking improvement on dystonia and pain. The duration of subjective improvement after completion of a combined protocol ranged from 2 to 6 months (3.4 ± 1.7), which was significantly longer than after cer-atDCS performed alone (*p* = 0.011).

Except for patient 3, all patients continued BTX injections, which were performed on the week before the cer-atDCS sessions in all cases. It is not possible to assess whether BTX injections played a synergistic role with cer-atDCS therapy (combined or not with Mot-Training) in the clinical improvement, which was observed even though the schedule and doses of BTX were kept similar to the pre-stimulation period, except in patient 5, for whom, because of the clear clinical improvement, the BTX injections were spaced from 2 to 3.5 months apart.

## Discussion

In this open-label pilot case series of patients with CD, the combination of Mot-Training with cer-atDCS produced a greater and more prolonged improvement in dystonia, compared to the application of cer-atDCS alone.

The mechanism of action of tDCS is not fully known (25). The delivered constant current modulates the excitability of neurons by modifying the axonal membrane potential (depolarization or hyperpolarization) but these currents are too weak to generate action potentials and therefore directly activate a neuronal circuit (26, 27), unlike what happens with rTMS. However, while the current generated by rTMS with a figure-of-8 coil remains relatively focal into the brain, the current generated by a bipolar tDCS montage has a very broad distribution, and potentially the effect of polarization of many neurons (even of glial cells) can produce an amplifying effect. Thus, tDCS can indisputably produce clinical effects in the short term lasting beyond the stimulation session, and also in a longer term, with sustainable changes in synaptic transmission and neuroplasticity effects that can result from repeated sessions.

In this pilot case series, CD was improved by 37% on average on the TWSTRS score after cer-atDCS performed alone, with a duration of subjective improvement ranging from 1 to 3 months. These results support the lasting effect of tDCS and the value of cerebellar targeting for neuromodulation of sensorimotor disorders (28). However, when cer-atDCS was combined with Mot-Training, clinical improvement reached 53% on average on the TWSTRS score, with a significantly longer duration of efficacy of 2 to 6 months. According to the concept of metaplasticity (29, 30), the additional effect of the combined strategy suggests a priming effect of tDCS that would modify the level of synaptic activity of certain neuronal circuits, placing the brain in a favorable state to boost long-term synaptic plasticity processes produced by Mot-Training.

In fact, the place of physiotherapy in the treatment of CD is not well defined, usually considered as an adjuvant therapy in addition to BTX injections (31–33). The physiotherapy program used here (Mot-Training) is a personalized rehabilitation program focused on the activation of anti-dystonic cervical muscles to alleviate their inhibition and integrate them into the correction of cervical posture and movements. As for neuromodulation, the repetition of Mot-Training sessions at short intervals can promote adaptative synaptic plasticity, especially if the sessions are maintained over a long period of time (6, 7, 23). In CD, instrumental techniques based on peripheral nerve stimulation or biofeedback (33–36) require a technical environment that is hardly compatible with long-term application. The same is true with rehabilitation techniques that involve too much physical intensity. The Mot-Training protocol (3) used here responds well to these feasibility criteria in terms of frequency and duration over time and therefore of clinical relevance for treating CD. It is also for this reason that it appeared to us to be ideal for combining with tDCS with a view to potentiating the clinical effects of these two therapies.

### Limitations

First, the main limitation of this report lies in the small sample size, which limits the representativeness of the results.

Second, our patients received an open-label treatment, which of course cannot rule out a placebo effect.

Third, the combined protocol had greater efficacy than tDCS alone, but in the absence of a control condition consisting of Mot-Training alone, it is not possible to know whether cer-atDCS has an additive or synergistic effect compared to that of Mot-Training.

Fourth, the methodology presents significant heterogeneity in terms of protocol applied in each patient. For example, the number of cer-atDCS sessions was not the same among the 5 participants, from one to five cer-atDCS sessions in the first phase and from one to 12 cer-atDCS sessions combined with Mot-Training in the second phase. The protocol should have been more standardized and not solely dependent on patients’ availability to return to the center, but this is a naturalistic proof-of-concept pilot study.

Fifth, heterogeneity also applies to the clinical profile, such as a variable age, between 40 and 79 years. There is also clinical variability regarding the dystonic pattern and disease severity exhibited by patients. Additionally, the series included a patient with suspected neuroleptic-induced dystonia, introducing a potential confounding factor.

Sixth, four out of five patients continued BTX injections during the experimental procedure, with varying injection intervals and without standardized time between procedures. This factor could have a significant impact on the results obtained, as discussed for the two previously reported cases of patients with CD who benefited from cer-atDCS (11). It is possible that the combined strategy could have restored a certain efficacy to the BTX injections and therefore that this also contributed to the clinical improvement through an additive or synergistic effect. Thus, a potentiating effect of neuromodulation techniques (rTMS or tDCS) with BTX injections could be expected for the treatment of CD or blepharospasm for example (12, 13, 37).

## Conclusion and perspectives

Although placebo effects cannot be excluded, this open-label case series suggests that the combination of cer-atDCS with Mot-Training would have a greater potential for clinical efficacy in terms of intensity and duration than either technique taken in isolation. In the context of CD, only one previous study (38) had shown a therapeutic benefit on the severity of dystonia symptoms of a non-invasive brain stimulation technique applied to the cerebellum (an rTMS protocol called intermittent theta-burst stimulation) combined with motor training for the neck and an implicit learning task. In our series, patients reported sustained clinical improvement for several weeks after repeated sessions of cer-atDCS at short intervals, suggesting potential value of this approach for the treatment of CD in daily practice. Furthermore, from the perspective of a clinical application, another favorable element is the fact that the practice of tDCS can be performed at home. As the rehabilitation protocol is also self-applicable by the patient, the combined therapeutic strategy could be entirely performed at home, after initial training of the patient in a hospital environment. This is an extremely important perspective both in terms of the clinical impact of this type of treatment and in terms of healthcare costs.

In conclusion, the results observed in this pilot case series justify considering a larger study comparing the results provided for the treatment of CD in the long term (6 to 12 months) by the application of cer-atDCS alone, Mot-Training alone, and cer-atDCS combined with Mot-Training, including a sham procedure for the stimulation part.

## Data availability statement

The raw data supporting the conclusions of this article will be made available by the authors, without undue reservation.

## Ethics statement

Ethical approval was not required for the studies involving humans because they correspond to retrospective research conducted on already available and anonymous data. The studies were conducted in accordance with the local legislation and institutional requirements. The participants provided their written informed consent to participate in this study. Written informed consent was obtained from the individual(s) for the publication of any potentially identifiable images or data included in this article.

## Author contributions

J-PB: Writing – review & editing, Writing – original draft, Validation, Supervision, Methodology, Investigation, Formal analysis, Data curation, Conceptualization. CC: Writing – review & editing, Investigation. TC: Writing – review & editing, Investigation. AS: Writing – review & editing, Investigation. ED: Writing – review & editing, Investigation. PD: Writing – review & editing, Investigation. JN: Writing – review & editing, Investigation. J-PL: Writing – review & editing, Writing – original draft, Validation, Supervision, Formal analysis. J-PN: Writing – review & editing, Writing – original draft, Validation, Supervision, Methodology, Investigation, Formal analysis, Data curation, Conceptualization.

## Supplementary material

SUPPLEMENTARY VIDEO S1 Clinical examination of patient 1. Segment 1: identification of the "corrective muscles" (antagonists) of the dystonic attitude including an antecollis associated with a laterocollis to the left and shoulder drop. Segment 2: the three motor training exercises: (1) pull the head slightly back and reduce hyperlordosis (double-chin exercise); (2) same exercise combined with rotation of the head and neck to the anti-dystonic side; (3) elevation of the shoulders and rotation the head and neck to the anti-dystonic side. Segment 3: significant improvement after five consecutive days of treatment combining cerebellar anodal tDCS and motor training exercises.
